# Early and mid-embryonic upregulation of chloride, calcium, and sodium transporter genes mark functional maturation of the chorioallantoic membrane in broiler embryos

**DOI:** 10.3389/fphys.2025.1652828

**Published:** 2025-08-28

**Authors:** Sadid Al Amaz, Suman Poudel, Rajesh Jha, Birendra Mishra

**Affiliations:** Department of Human Nutrition, Food and Animal Sciences, College of Tropical Agriculture and Human Resilience, University of Hawaiʻi at Manoa, Honolulu, HI, United States

**Keywords:** broiler, embryogenesis, gene expression, ion transporter, transcriptomics

## Abstract

The chorioallantoic membrane (CAM) is essential for ion transport, acid-base homeostasis, and respiratory gas exchange during chicken embryonic development. The temporal expression of ion transporter-related genes in CAM is still inadequately investigated. This study examined the developmental regulation of genes associated with the transport of calcium (Ca), sodium (Na), bicarbonate (HCO_3_^−^), potassium (K), protons (H^+^), and chloride (Cl^−^) in the CAM at embryonic days (ED) 12, 15, and 18. Six hundred fertilized Cobb 500 broiler eggs were obtained from a local hatchery. After candling, on ED 10, a total of 236 eggs were selected for this experiment. All the eggs were incubated in an automated temperature regulation of 37.5°C, 55% relative humidity (RH), and egg rotation every 2 h. On days 12, 15, and 18 of the embryonic period, CAM tissues were collected (n = 6 per group), total RNA isolated, and target genes were analyzed using qPCR. At ED 12, chloride transporter genes (*CLCN2, SLC4A1,* and *SLC26A9*) were significantly upregulated compared to ED 18 (*P* < 0.05). At ED 15, calcium transporters (*ATP2A2, ATP2A3, ITPR1, ATP2B4, SLC8A1, SLC8A3, TRPV6,* and *RYR1*) were significantly upregulated compared to ED 12 and/or ED 18 (*P* < 0.05). Sodium transporters (*ATP1A1* and *SCNN1B*), bicarbonate transporters (*SLC4A4* and *SLC4A10*), potassium transporters (*KCNMA1)*, and proton transporters (*CA7* and *CA9*) were also significantly upregulated at ED 15 compared to ED 12 (*P* < 0.05). At ED 18, sodium transporter (*ATP1B1, SCNN1A,* and *SGK1*) and proton transporter (*CA2*) were significantly upregulated compared to ED 12 and/or ED 15 (*P* < 0.05). The results indicate a synchronized, stage-dependent transcriptional activation of ion transporter genes in the CAM, especially at the mid-embryonic stage.

## 1 Introduction

The partial fusion of the chorion and allantois forms the corioallointoic membrane (CAM) between embryonic days (ED) 5 and 6 (Melkonian et al., 2002). This structure develops incrementally, adhering to the acellular inner shell membrane located directly beneath the eggshell, and by ED 11 to 12, it completely encases the embryo and the residual egg contents (Gabrielli and Accili, 2010). The chick CAM is a fundamental extraembryonic membrane that performs several functions during embryonic development, including vasculature development (collagens, integrins, and laminins), respiratory gas exchange (annexins, hemoglobins, and tubulins), calcium ion (ATPases, CaBPs, and carbonic anhydrases) transport from the eggshell, embryonic acid-base homeostasis, ion and H_2_O reabsorption from the allantoic fluid, and chemical defense against infectious pathogens (cystatins, histones, and serpins) (Gabrielli and Accili, 2010; Ahmed et al., 2022).

Despite the immense importance of CAM during embryogenesis, gene expression related to ion transportation, acid-base balance, and gaseous exchange has not been widely investigated. Therefore, considering the importance, we hypothesize that the ion transportation, acid-base balance, and gaseous exchange-related gene expression would be upregulated in the mid-embryonic stage as the CAM is functionally activated by ED 12. So, this study aims to investigate the Ca (*ATP2A2, ATP2A3, ATP2B1, ATP2B2, ATP2B4, ITPR1, RYR1, TRPV6, SLC8A1,* and *SLC8A34*), Na (*ATP1A1, ATP1B1, SCNN1A, SGK1,* and *SCNN1B*), HCO_3_^−^ (*SLCA4, SLC4A5, SLC4A7, SLC4A8,* and *SLC4A10*), K (*KCNJ15, KCNJ16,* and *KCNMA1*), proton (*CA2, CA4, CA7,* and *CA9*), and Cl (*CLCN2, CLCN5*, *SLC4A1, SLC26A9,* and *CFTR*) ion transporter-related genes expression at ED 12, ED 15 and ED 18 of embryonic period.

We selected ED 12, ED 15, and ED 18 to represent key transitional and functional stages in CAM development—specifically the shift from vascularization (ED 12) to peak ion transport and respiratory function (ED 15), and finally to intensive mineral mobilization and osmoregulation prior to hatching (ED 18) (Givisiez et al., 2020). By analyzing multiple ion transporters and acid-base regulatory genes, we aimed to assess the coordinated molecular network underlying CAM functions, such as calcium mobilization for skeletal development (Halgrain et al., 2022), bicarbonate and chloride exchange for pH homeostasis (Everaert et al., 2008), and fluid/electrolyte transport is crucial for embryonic viability (Hamburger and Hamilton, 1992). These genes work synergistically to ensure proper embryonic development, particularly during the critical pre-hatch phase.

## 2 Materials and methods

### 2.1 Incubation

All animal experimentation followed the regulations approved by the Institutional Animal Care and Use Committee of the University of Hawaii (Approval No. 17-2605-6). This research utilized animal experimentation from our previous studies (Al Amaz et al., 2024a). Briefly, six hundred fertilized Cobb 500 broiler eggs were procured from Asagi Hatchery Inc. (Honolulu, HI). The eggs were randomly allocated among three incubators (GQF Incubator, Savannah, GA), each containing 200 eggs, despite the total capacity of the incubators being 270 eggs. Following candling at ED 10, a total of 474 eggs with viable embryos were chosen for the experiment. From there, 236 eggs were selected for this experiment and transferred to one incubator. The remaining embryos were used for other experiments (Al Amaz et al., 2024a; Al Amaz et al., 2025a; Amaz et al., 2024b; Al Amaz et al., 2024c). All the eggs were incubated according to the standard protocol, which involved automated temperature control at 37.5°C and 55% relative humidity (RH), as well as egg rotation every 2 h.

### 2.2 Sample collection

The CAM tissues were collected on ED 12, ED 15, and ED 18 (n = 6/group). The eggs were initially broken into petri dishes, followed by the euthanization of the embryos through carbon dioxide asphyxiation for sampling purposes. The CAM tissue was collected, rapidly snap-frozen, and stored at −80°C until RNA extraction.

### 2.3 Quantitative real-time PCR (qPCR)

Total RNA was extracted from CAM tissue using TRIzol reagent (Invitrogen, Carlsbad, CA) according to the manufacturer’s instructions. The RNA concentration was quantified using a NanoDrop™ spectrophotometer (ThermoFisher Scientific, Madison, WI) by measuring purity (A260/280∼2.0). The quality of the RNA was determined by running samples on 2% agarose gel. On the gel, high-quality RNA showed two distinct rRNA bands (28S and 18S), with the 28S band approximately twice as intense as the 18S band. The absence of smearing indicated minimal degradation, confirming the RNA was intact and suitable for downstream applications. Then, the RNAs were reverse transcribed into complementary DNA (cDNA), and the target genes were analyzed using qPCR following the established protocol as previously described (Chaudhary et al., 2023; Amaz et al., 2025b). The primer sequences utilized for gene expression analysis are enumerated in Table 1. The NCBI Primer-Blast tool created particular primer pairs for each gene’s detection. A High-Capacity cDNA Reverse Transcription Kit (Applied Biosystems, Foster City, CA) was used to reverse-transcribe 1 μg of total RNA (20 μL of reverse transcription reaction mixture) into cDNA, which was subsequently diluted with nuclease-free water (1:25). qPCR was carried out using the PowerUp SYBR Green Master Mix (Applied Biosystems, Foster City, CA) and real-time PCR equipment (Applied Biosystems). To achieve a final reaction volume of 10 μL, the qPCR reaction mixture included 3 μL of cDNA, 5 μL of PowerUp SYBR Green Master Mix, and 1 μL of each forward and reverse primer at a concentration of 5 μmol. The qPCR reaction was conducted using the standard cycling mode. The qPCR thermal profile consists of an initial denaturation at 95°C for 2 min, followed by 40 cycles of denaturation at 95°C for 15 s and annealing/extension at 60°C for 30 s. A melting curve analysis was conducted to validate the SYBR Green-based amplicon. Furthermore, the specificity of each primer pair was evaluated through 1% gel electrophoresis of the qPCR products. The analysis was performed in triplicate for three housekeeping genes: *GAPDH*, *β-actin*, and *TBP*. The *TBP* expression was consistently stable across the CAM tissues. Post-amplification, the cycle threshold (Ct) values were documented, and gene expression levels were determined utilizing *TBP* as the reference gene, following the 2^−ΔΔCT^ method (Livak and Schmittgen, 2001).

**TABLE 1:** List of 35 primers used in qPCR.

Gene	Accession no.	Primer sequence (5′ to 3′)
*TBP* (TATA-box binding protein)	XM_025148547.3	F: TAGCCCGATGATGCCGTAT
		R: GTTCCCTGTGTCGCTTGC
*GAPDH* (Glyceraldehyde-3-phosphate dehydrogenase)	NM_204305.2	F: AGCTTACTGGAATGGCTTTCCG
		R: ATCAGCAGCAGCCTTCACTACC
*B-Actin* (Beta-actin)	NM_205518.2	F:GAG AAA TTG TGC GTG ACA TCA
		R: CCT GAA CCT CTC ATT GCC A
*ATP2A2* (ATPase sarcoplasmic/endoplasmic reticulum Ca^2+^ transporting 2)	XM_415130	F: GCAGCTTGCATATCTTTTGTGCTG
		R: CATTTCTTTCCTGCCACACTCC
*ATP2A3* (ATPase sarcoplasmic/endoplasmic reticulum Ca^2+^ transporting 3)	NM_204891	F: CAACCCCAAGGAGCCTCTTATC
		R: GGTCCCTCAGCGTCATACAAGAAC
*ATP2B1* (ATPase plasma membrane Ca^2+^ transporting 1)	XM_416133	F: CTGCACTGAAGAAAGCAGATGTTG
		R: GCTGTCATATACGTTTCGTCCCC
*ATP2B2* (ATPase plasma membrane Ca^2+^ transporting 2)	XM_001231767	F: TTACTGTACTTGTGGTTGCTGTCCC
		R: GGTTGTTAGCGTCCCTGTTTTG
*ATP2B4* (ATPase plasma membrane Ca^2+^ transporting 4)	XM_418055	F: GCTGGTGAAGTTGTCATCCGTC
		R: TGCTCTGAAGAAAGCTGATGTTGG
*ITPR1* (Inositol 1,4,5-trisphosphate receptor type 1)	XM_414438	F: AATGGCAAAAGGCGAGGAAAGC
		R: GGAGCAGCAGCAAGCGGG
*RYR1* (Ryanodine receptor 1)	X95,266	F: GTTCCTCTGCATCATCGGCTAC
		R: AATTGCTGGGGAAGGACTGTG
*TPRV6* (Transient receptor potential cation channel subfamily V member)	XM_416530	F: AACACCTGTGAAGGAGCTGGTGAG
		R: TCTGCTGCTTGTTTTGTTGCC
*SLC8A1* (Solute carrier family 8 member A1)	NM_001079473	F: GGATTGTGGAGGTTTGGGAAGG
		R: CTGTTTGCCAGCTCGGTATTTC
*SLC8A3* (Solute carrier family 8 member A3)	XM_001231413	F: GGAGAGACCACAACAACAACCATTC
		R: AGCTACGAATCCATGCCCACAC
*ATP1A1* (ATPase Na^+^/K^+^ transporting subunit alpha 1)	NM_205519.1	F: GCTGTGGGTCAATCTGGTGA
		R: GATAGGCTGTTGAGGGCGTT
*ATP1B1* (ATPase Na^+^/K^+^ transporting subunit beta 1)	XM_416133	F: CTGCACTGAAGAAAGCAGATGTTG
		R: GCTGTCATATACGTTTCGTCCCC
*SCNN1A* (Sodium channel epithelial 1 alpha subunit)	NM_205145	F: GCTTGCCAGAAAACAGTCCCTC
		R: AGTCAGACTCATCCAGGTCTTTGG
*SCNN1B* (Sodium channel epithelial 1 beta subunit)	XM_425247	F: ATGGAAGTAGACCGCAGT
		R: GTTGTATGGCAGCACAGT
*SGK1* (Serum/glucocorticoid regulated kinase 1)	XM_040664661.2	F: GCCCAGTCCATCACAACAGA
		R: ATGCCGTGCAAGAAGAACCT
*SLC4A4* (Solute carrier family 4 member 4)	XM_420603	F: GGAAAGCACCATTCTTCGCC
		R: CCTCCAAAAGTGATAGCATTGGTC
*SLC4A5* (Solute carrier family 4 member 5)	XM_423797	F: TGAACGTCTCCGCTACATCCTG
		R: ACTTTATCCACCTGGCTGACTCC
*SLC4A7* (Solute carrier family 4 member 7)	XM_418757	F: AAATTGCCAAGTTCGTGGTGG
		R: GCGAAGCAAATGAGAAGTTACGG
*SLC4A8* (Solute carrier family 4 member 8)	XM_001232427	F: AGAAGAAGAAGTTGGACGATGCC
		R: GGTCAGTTCTGTCCTTGCTGTTCTG
*SLC4A10* (Solute carrier family 4 member 10)	XR_026836	F: CGCTGATGACAGATGAGGTGTTC
		R: GGTGGTTCTATTCGGATTGTTGG
*KCNJ15* (Potassium inwardly rectifying channel subfamily J member 15)	XM_425554.8	F: TGAGGGAAGGGAGACTCTGA
		R: GCTTCCATCCTCACTGCTTC
*KCNJ16* (Potassium inwardly rectifying channel subfamily J member 16)	BI394769	F: CATTCCTGTTCTCCCTGGAA
		R: CATTTTAGCCAAGGCTGCTC
*KCNMA1* (Potassium calcium-activated channel subfamily M alpha 1)	M87294	F: GGGATGATGCGATCTGTCTT
		R: GACAAACCCACAAAGGCACT
*CA2* (Carbonic anhydrase II)	NM_001398092.1	F: ATCGTCAACAACGGGCACTCCTTC
		R: TGCACCAACCTGTAGACTCCATCC
*CA4* (Carbonic anhydrase IV)	NM_001361182.2	F: GCTAACACATTTTTCCCCCTTCC
		R: CTTTATAGCACATCGCATCAGCC
*CA7* (Carbonic anhydrase VII)	XM 425734.4	F: GCACAAGTCTTATCCCATTGCC
		R: GCCGTTGTTGGAGATGTTGAGAG
*CA9* (Carbonic anhydrase IX)	XM 425740.3	F: CCTGACAACCTGCACCTCTA
		R: GAGGTGGTTGTCGTCTGTCT
*CLCN2* (Chloride voltage-gated channel 2)	NM_001031514	F: CCTGGACACCAATGTGATGCTG
		R: CACGAAGGTCTTCAGGGTGAGATAC
*CLCN5* (Chloride voltage-gated channel 5)	NM_001195795	F: CGATTGGAGGAGTGCTCTTTAGTC
		R: CAAAAGGATTGATGGAACGCAG
*SLC4A1* (Solute carrier family 4 member 1)	AB056748	F: TGAGACCTTCGCCAAACTCG
		R: TTCAGCTTCTGCGTGTAGGT
*SLC26A9* (Solute carrier family 26 member 9)	NM_001001741	F: GCCTCTTCGATGAGGAGTTTGAG
		R: CTGACCCCACCAAGAACATCAG
*CFTR* (Cystic fibrosis transmembrane conductance regulator)	NM_001105666	F: AAGAGGGCAGGGAAGATCAACGAG
		R: CGGGTTAGCTTCAGTTCAGTTTCAC

F = Forward primer, R = Reverse primer; the primer sequences were created with NCBI primer-BLAST.

### 2.4 Statistical analysis

The gene expression was evaluated utilizing GraphPad (GraphPad Software, San Diego, CA). After conducting a one-way analysis of variance (ANOVA), the Tukey-HSD test was employed to compare the means of the different treatment groups. All data are expressed as mean ± Standard error of the mean (SEM). SEM represents the variability of the sample mean and is calculated as the standard deviation (SD) divided by the square root of the sample size (n). The threshold for statistical significance was established at *P* < 0.05.

## 3 Result

### 3.1 Calcium (Ca) transporter-related gene expression

**FIGURE 1:**
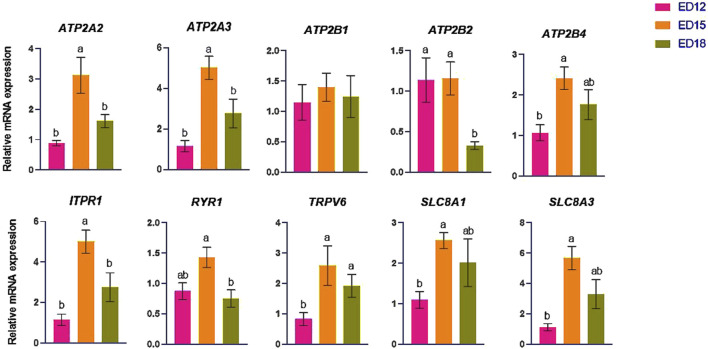
Changes in calcium ion transporter genes during embryonic days (ED) in the Corioallionic membrane of broiler embryos (n = 6/group). Data presented as mean ± SEM. Different letters indicate a significant difference (P < 0.05) among the age groups. No letters means no significant difference.

The expression pattern of the Ca transporter-related genes (*ATP2A2, ATP2A3, ATP2B1, ATP2B2, ATP2B4, ITPR1, RYR1, TRPV6, SLC8A1,* and *SLC8A3*) in the CAM were summarized in Figure 1. The expressions of *ATP2A2, ATP2A3,* and *ITPR1* were significantly increased at ED 15 compared to the other days (*P* < 0.05). *ATP2B4*, *SLC8A1,* and *SLC8A3* were significantly lower at ED 12 than at ED 15 (*P* < 0.05), while *TRPV6* was significantly lower at ED 12 compared to the other days (*P* < 0.05). *ATP2B2* was significantly lower at ED 18 compared to the other days (*P* < 0.05), while *RYR1* was significantly lower at ED 18 compared to ED 15 (*P* < 0.05). *ATP2B1* expression remained unchanged across the time points.

### 3.2 Sodium (Na) transporter-related gene expression

**FIGURE 2:**
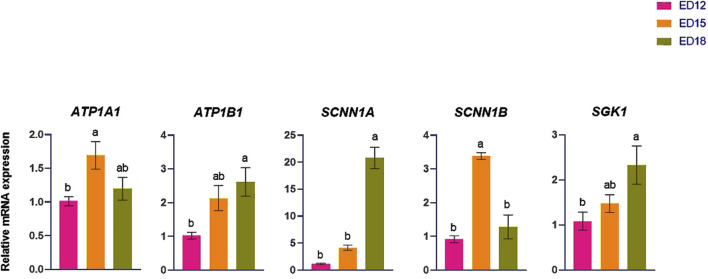
Changes in sodium ion transporter genes during embryonic days (ED) in the Corioallionic membrane of broiler embryos (n = 6/group). Data presented as mean ± SEM. Different letters indicate a significant difference (P < 0.05) among the age groups. No letters means no significant difference.

The Na transporter-related genes (*ATP1A1, ATP1B1, SCNN1A, SGK1,* and *SCNN1B*) are shown in Figure 2. *ATP1A1* was significantly higher at ED 15 than in ED 12 (*P* < 0.05), while *SCNN1B* was significantly higher at ED 15 compared to the other groups (*P* < 0.05). *ATP1B1* and *SGK1* were significantly higher at ED 18 than at ED 12 (*P* < 0.05). *SCNN1A* was significantly higher at ED 18 compared to the other groups (*P* < 0.05).

### 3.3 Bicarbonate (HCO_3_^−^) transporter-related gene expression

**FIGURE 3:**
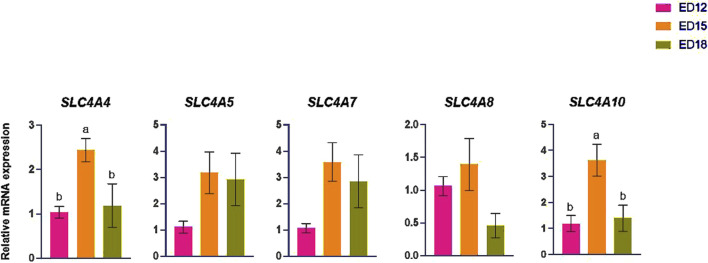
Changes in bicarbonate ion transporter genes during embryonic days (ED) in the Corioallionic membrane of broiler embryos (n = 6/group). Data presented as mean ± SEM. Different letters indicate a significant difference (P < 0.05) among the age groups. No letters means no significant difference.

The HCO_3_^−^ -transporter-related genes (*SLCA4, SLC4A5, SLC4A7, SLC4A8,* and *SLC4A10*) are shown in Figure 3. *SLCA4* and *SLC4A10* were significantly higher at ED 15 compared to ED 12 and ED 18 (*P* < 0.05). *SLC4A5*, *SLC4A7,* and *SLC4A8* expression were not changed at any time points.

### 3.4 Potassium (K) and proton (H^+^) transporter-related gene expression

**FIGURE 4:**
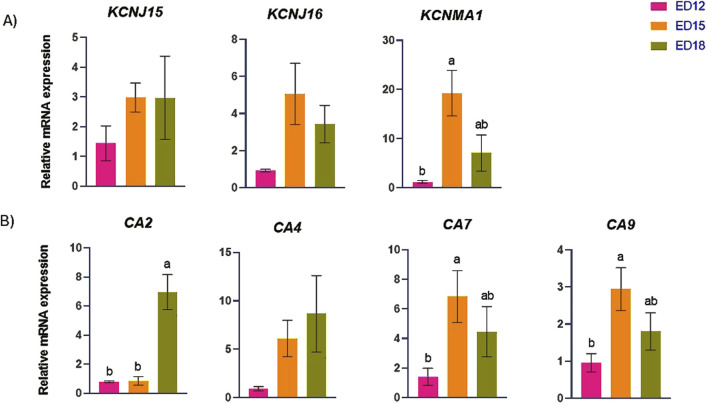
Changes in **(A)** potassium and **(B)** proton transporter genes during embryonic days (ED) in the Corioallionic membrane of broiler embryos (n = 6/group). Data presented as mean ± SEM. Different letters indicate a significant difference (P < 0.05) among the age groups. No letters means no significant difference.

The K transporter-related genes (*KCNJ15, KCNJ16,* and *KCNMA1*) and H^+^ transporter-related genes (*CA2, CA4, CA7,* and *CA9*) are presented in Figures 4A,B. *KCNMA1* was significantly higher at ED 15 compared to ED 12 (*P* < 0.05). *CA2* was significantly higher at ED 18 than in the other groups (*P* < 0.05). *CA7* and *CA9* were significantly higher at ED 15 compared to ED 12 (*P* < 0.05). *KCNJ15, KCNJ16,* and *CA4* were not changed at ED12,15 nad 18.

### 3.5 Chlorine (Cl) transporter-related gene expression

**FIGURE 5:**
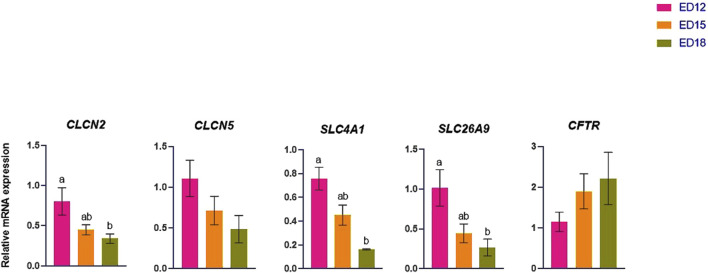
Changes in chloride ion transporter genes during embryonic days (ED) in the Corioallionic membrane of broiler embryos (n = 6/group). Data presented as mean ± SEM. Different letters indicate a significant difference (P < 0.05) among the age groups. No letters means no significant difference.

The Cl transporter genes (*CLCN2, CLCN5*, *SLC4A1, SLC26A9,* and *CFTR*) are presented in Figure 5. *CLCN2*, *SLC4A1,* and *SLC26A9* were significantly higher at ED 12 than at ED 18 (*P* < 0.05). *CLCN5* and *CFTR* remained unchanged across the time points.

## 4 Discussion

### 4.1 Calcium (Ca) ion transportation

Calcium is reabsorbed from the eggshell and transported to the embryo for bone development throughout the embryonic period (Johnston and Comar, 1955). Transporting calcium from the eggshell into the embryonic circulation requires Ca^2+^-activated ATPase. This membrane-bound enzyme, which necessitates ATP and Mg^2+^ and is activated by Ca^2+^, is specifically situated within the CAM ectoderm, the layer adjacent to the shell. Its activity markedly escalates during phases of swift calcium accumulation, underscoring its significance in skeletogenesis (Tuan and Knowles, 1984). In this study, *ATP2A2* and *ATP2A3* were significantly higher at ED 15 than at the other time points, while *ATP2B4* was significantly higher at ED 15 than at ED 12. However, *ATP2B2* was significantly higher at ED 15 and ED 12 than at ED 18. Taken together, it is evident that the peak initiation of calcium transportation happens at ED 12 and then peaks at ED 15 during bone mineralization. This coincides with the known period of active calcium mobilization and mineralization of the embryonic skeleton, which has been documented to intensify between ED12 and ED16 in chickens (Halgrain et al., 2022). IP3R1 encodes *ITPR1*, a member of the IP3 receptor family (IP3R1–3), and is essential for intracellular calcium signaling because it releases calcium ions into the cytosol from the endoplasmic reticulum (Sun et al., 2025). *ITPR1* was significantly higher at ED 15 than in the other groups. The *RYR1* codes ryanodine receptor 1, a protein essential for the release of calcium and contraction of skeletal muscle (Alvarellos et al., 2016). Significantly higher *ITPR1* and *RYR1* expressions at ED 15 indicate heightened intracellular calcium signaling. *TRPV6* is crucial in initiating calcium transport in CAM (Huang et al., 2022). The *SLC8A1* and *SLC8A3*, belonging to the solute carrier family 8, encode sodium/calcium exchangers in the plasma membrane. These exchangers function bidirectionally, crucially contributing to calcium homeostasis by facilitating the exchange of intracellular Ca^2+^ and extracellular Na^+^ ions (Gabellini et al., 2002). *TRPV6, SLC8A1,* and *SLC8A3* were significantly higher at ED 15 than ED 12, allowing increased transcellular calcium influx and efflux.

### 4.2 Sodium (Na) ion transporation

The coordinated expression of Na^+^ ion transport genes precisely controls the regulation of sodium homeostasis in the embryonic CAM. The *ATP1A1* is essential for cation transport and generates and maintains the Na+/K+ ion electrochemical gradients throughout the cell membrane (Lin et al., 2021). In this study, *ATP1A1* was significantly higher at ED 15 than at ED 12, suggesting enhanced sodium transport activity during mid-embryonic development. The Epithelial Sodium Channel consists of α, β, and γ subunits, encoded by the *SCNN1A*, *SCNN1B*, and *SCNN1G*, corresponding to the sodium channel epithelial 1 subunits α, β, and γ, respectively (Pierandrei et al., 2021). *SGK1* regulates sodium retention, potassium excretion, and mineral and nutrient transport across renal, gastric, and intestinal tissues. It also influences salt appetite, blood coagulation, blood pressure, and insulin-dependent glucose uptake (Lang et al., 2006). *SGK1* and *SCNN1A* were significantly higher at ED 18 than ED 12, indicating their role in the terminal phase of ENaC activation and effective sodium absorption in anticipation of hatching, when the demand for sodium transport is at its highest. However, *SCNN1B* was significantly higher at ED 15 than at the other time points. *SCNN1B* may facilitate channel assembly at an earlier stage, whereas *SCNN1A* may serve as a rate-limiting factor for the complete functionality of ENaC, which becomes essential as hatching approaches when the embryo necessitates optimal ion and fluid homeostasis. This temporal separation facilitates the sodium transport pathway’s synchronized accumulation and activation.

### 4.3 Bicarbonate (HCO_3_^−^) ion transportation

The solute carrier family 4 is a crucial protein that mediates various physiological processes, including molecular transduction, protein-to-protein interactions, and the transport of different ions across the cell membrane (Zhong et al., 2023). In chickens, *SLC4A4* encodes the electrogenic sodium bicarbonate cotransporter 1. This protein is essential for transporting sodium and bicarbonate ions across cellular membranes (Bahadoran et al., 2018). Acid extrusion is mediated by *SLC4A10*, which explicitly uses the transmembrane gradient of Na^+^ to promote cellular net uptake of HCO_3_^−^ (Maroofian et al., 2024). *SLC4A4* and *SLC4A10* were significantly upregulated at ED 15 compared to the other time points, signifying increased bicarbonate transport and pH buffering during this phase. In neuronal and epithelial tissues, *SLC4A8* encodes the Na^+^-driven Cl^−^/HCO_3_^−^ exchanger, which couples sodium influx with chloride–bicarbonate exchange to regulate intracellular pH and secrete bicarbonate (Remigante et al., 2022). SLC4A5 is a transmembrane protein that operates as an electrogenic cotransporter, facilitating the concurrent transport of HCO_3_^−^ and Na^+^ ions in the same direction (Stütz et al., 2009). The *SLC4A5*, *SLC4A7,* and *SLC4A8* showed numerically higher expression at ED 15 compared to the other time points, bolstering the higher bicarbonate-transporting activity during the mid-embryonic phase. These findings underscore a stage-specific activation of bicarbonate transport mechanisms, presumably to accommodate higher metabolic demands and preserve intracellular pH equilibrium during accelerated embryonic development. This aligns with previous studies indicating that embryonic metabolic rate and CO_2_ production significantly increase during this window, requiring tight regulation of intracellular pH (Tazawa, 1980).

### 4.4 Potassium (K) and proton (H^+^) transportation

Potassium voltage-gated channel subfamily J member 16 (KCNJ16) protein encodes the inward rectifier potassium channel Kir5.1, dimerizes with Kir4.2 (encoded by KCNJ15) and Kir4.1 (encoded by KCNJ10) to form functional heteromeric channels that help control membrane potential and potassium ion transport (Zhang et al., 2021). The “Big K+” (BK) large-conductance calcium and voltage-activated K+ channel’s pore-forming α subunit is encoded by KCNMA1. Both excitable and non-excitable cells are found in tissues with a large distribution of BK channels (Bailey et al., 2019). In this study, *KCNJ15* expression remained stable across developmental stages, suggesting a constitutive role in baseline K^+^ conductance that may not require transcriptional regulation during this period. In contrast, *KCNJ16* showed a trend toward upregulation at ED15, which may reflect a supportive role in enhancing Na^+^/K^+^-ATPase function or accommodating increased ion exchange demands as CAM ion transport activity peaks. This subtle change may indicate post-transcriptional regulation or gradual recruitment of K^+^ channels to meet the increased ionic flux during mid-embryogenesis. However, *KCNMA1* was significantly upregulated at ED 15 compared to ED 12. This upregulation indicates a developmentally regulated activation of BK channels, presumably facilitating rapid cellular activity, volume regulation, or signal transduction during the mid-embryonic phase. Carbonic anhydrases (CAs) are widespread metalloenzymes that facilitate the crucial reaction of converting carbon dioxide (CO_2_) and water (H_2_O) into bicarbonate (HCO_3_^−^) and protons (H^+^) (Occhipinti and Boron, 2019). The calcium carbonate (CaCO_3_) is broken down into Ca^2+^ and HCO_3_^−^ by the protons generated by CA2, which acidify the region surrounding the eggshell. Specialized cells in the CAM subsequently absorb the released calcium ions and transfer them to the growing chick embryo (Huang et al., 2022). The *CA7* may enhance acid-base equilibrium and CO_2_ transport in the CAM, which is essential for embryonic gas exchange and pH regulation (Supuran, 2008). The transmembrane enzyme CA9 catalyzes the reversible hydration of CO_2_ to bicarbonate and protons, preserving pH homeostasis in hypoxic environments (Swietach et al., 2010). In this study, *CA2* was significantly upregulated at ED 18, whereas *CA7* and *CA9* were significantly upregulated at ED 15 compared to ED 12. The initial increase in *CA7* and *CA9* suggests an upregulation of respiratory and pH homeostasis facilitation. In contrast, the subsequent induction of *CA2* is associated with calcium extraction for osseous development, indicating a sequential functional transition in CA isozyme activity as embryogenesis progresses.

### 4.5 Chloride (Cl^−^) ion transporation

A voltage-gated chloride channel (CLC-2), widely expressed and distributed throughout the body, is encoded by chloride voltage-gated channel 2 (CLCN2). This encoded transmembrane protein is essential for electrogenesis, homeostatic cell volume regulation, and ion gradient maintenance. It also stabilizes chloride ion homeostasis in various cells (Thiemann et al., 1992). The voltage-gated chloride channel (ClC-5) encoded by CLCN5 mainly involves ion transport and endosomal acidification, controlling vesicular trafficking and electrolyte balance in the epithelium (Jentsch and Pusch, 2018). In this study, *CLCN2* was significantly upregulated at ED 12 compared to ED 18, suggesting an aid in the electrogenesis and initial stabilization of chloride ions during the early stages of vascular and epithelial growth and development in CAM. In erythrocytes and epithelial tissues, anion exchanger 1, a transmembrane protein that facilitates chloride–bicarbonate exchange and is necessary for CO_2_ transportation and pH regulation, is encoded by *SLC4A1* (Alper, 2006). *SLC26A9* either physically or functionally interacts with the CFTR chloride channel to control CFTR activity or is controlled by CFTR (Remigante et al., 2022). *SLC4A1* and *SLC26A9* were significantly decreased from ED 12 to ED 18. The decrease in ED 18 expression indicates a developmental shift from chloride-driven acid-base regulation as mineral demands and calcium mobilization become more important in later stages.

## 5 Conclusion

**FIGURE 6:**
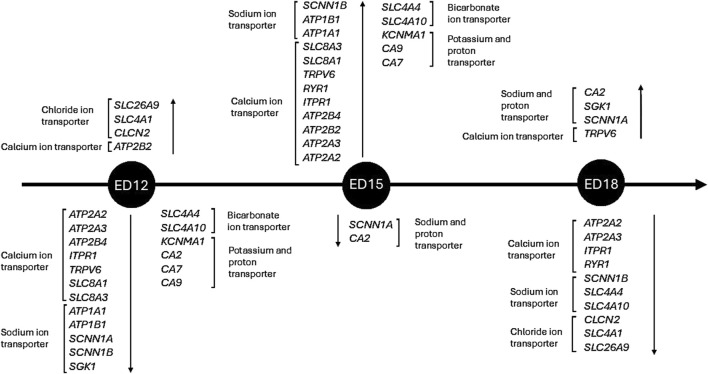
Timeline of ion transportation-related genes in Corioallionic membrane development. Only significant differences (*P < 0.05*) are considered in the timeline.

This study demonstrates that the embryonic CAM’s ion transport and pH-regulating systems are activated in a tightly controlled, stage-specific manner. Early development is marked by improved gas exchange and chloride-driven acid-base balance, driven by chloride, which promotes epithelial growth and CO_2_ elimination (Figure 6). Increased ion mobilization, especially of calcium and sodium, is seen in the mid to late stages to promote fluid homeostasis and bone formation. The temporal separation of calcium/sodium transport and chloride-mediated pH regulation ensures adequate metabolic support, mineral delivery, and physiological preparation for hatching. This study reveals that the CAM acts as a dynamic interface incorporating developmental demands, acid-base regulation, and ion transport. Our findings provide a strong foundation for conducting functional validation studies. Future efforts will focus on localizing proteins and quantifying them at various stages of embryonic development to determine if changes in gene expression are correlated with protein activity. Functional experiments, such as ion flux measurements and pharmacological inhibition, will clarify the physiological significance of these genes. Integrating transcriptome data with metabolic, physiological, and morphological outcomes will ultimately enhance our understanding of how CAM maturation supports embryo survival and development. This information may also improve poultry breeding practices for selecting future breeding flocks and enhance biomedical models that utilize the CAM.

## Data Availability

The datasets presented in this study can be found in online repositories. The names of the repository/repositories and accession number(s) can be found in the article/supplementary material.
